# The War Office and the Poor-Law Infirmary

**Published:** 1916-03-25

**Authors:** 


### THE WAR OFFICE AND THE POOR-LAW INFIRMARY.

### Military and Civilian Standards.

One interesting effect of the war has been the extensive use made by the War Office of Poor-Law infirmaries for the treatment of sick and wounded soldiers. Within six months of the outbreak of hostilities it became obvious that the accommodation at the existing military hospitals as well as in the improvised Territorial hospitals and that which could be provided by the voluntary hospitals was hopelessly inadequate to meet the requirements of our greatly enlarged Army. The urgency of the need made it impossible to seek a solution of the difficulty by the erection of new hospitals, and it became necessary to find suitable buildings which could be taken over at once by the military authorities. It is to the credit of many Boards of Guardians that early in the war they had realised the possible necessity for the provision of additional accommodation for our wounded, and had offered to the War Office unoccupied wards in their infirmaries. These offers were at first politely refused, but no doubt they suggested to the headquarter staff of the R.A.M.C. an easy means of solving the difficulties in which they soon found themselves involved. At any rate, early in 1915, after consultation with the Treasury and the Local Government Board, the War Office approached various Boards of Guardians, both in London and the provinces, and asked them to hand over temporarily their infirmaries to the R.A.M.C. In practically all cases the Guardians at once agreed and transferred their institutions, together with the staff and equipment, to the War Office. Now that a year has elapsed since the first of these transfers took place, it is of interest to consider the changes the new authority found it necessary to make so that treatment could be efficiently carried out and the wounded and sick restored quickly to health. It is of the greater interest to do so because Poor-Law Guardians and officials of the Local Government Board have so frequently boasted of recent years that the Poor-Law infirmary is in every respect as well equipped as the voluntary hospital. In testing the value of these assertions by the changes made by the War Office it must be remembered that the infirmaries taken over were carefully selected, and only those known to be well equipped as judged by the Poor-Law standard were chosen. The difference in the nature of the cases admitted under the new conditions must also be borne in mind. An increase in the equipment and staff for operative surgery, in the provision for massage and mechano- and electro-therapy, and in the *x*-ray department was a necessary corollary of this change in the type of case, and casts no slur on the Guardians. On the other hand, the provision for treatment was simplified by the exclusion of certain special classes of patients, such as the gynæcological and obstetrical cases, and the children formerly admitted to the infirmary. The work of the medical and nursing staff is also lightened by the War Office requirements that patients on their discharge should be fit for duty, or light duty, so that the wards of a war hospital contain many convalescent patients who under the ordinary conditions of a Poor-Law infirmary would be treated in the out-door dispensary.

Bearing these considerations in mind, we are now in a position to appreciate the extent of the deficiency as regards staff and equipment for the proper treatment of patients the War Office found existing in these selected infirmaries. It may be said at once that the structural arrangements of the wards and, in most cases, of the operating unit, were found to be admirable and to require little or no alteration. The wards were also well equipped, but the stock of surgical instruments, of *x*-ray apparatus, of appliances for bacteriological and pathological work, and for the more specialised methods of clinical examination and of treatment, had to be very considerably increased. The amount of the additions required under these headings varied considerably in different infirmaries, and in some no provision under one or more of them existed at all. It was, however, when the medical staff was considered that the most serious defects were discovered. This bears out a criticism which has been frequently made in The Hospital—that Guardians will provide buildings and equipment but fail to realise the importance of a medical staff adequate to make full use of them. In order to compare the Guardians' with the War Office standard in this respect let us take a 500-bed unit. The Guardians would staff this with one medical superintendent and two or, in the case of the most liberal-minded boards, three junior medical officers. The War Office staff would consist of the officer-in-charge, the registrar, ten medical men, of whom four would be full-time and six half-time, a radiographer, an anæsthetist, a pathologist, an ophthalmic surgeon, and a dental surgeon. Another difference is found in the character of the staff. In the case of the Guardians the medical superintendent would be usually selected on account of his administrative ability, the senior assistant medical officer would be a man who had had a few years' experience as a resident in infirmaries or hospitals, and the remainder would be recently qualified men. On the other hand, most of the War Office staff would be men of considerable experience in their profession, and would be chosen so as to include a certain number of men who had devoted themselves to operative surgery. In addition, in London at any rate, the War Office has organised a consulting staff of ophthalmic surgeons, specialists in diseases of the ear, throat, and nose, alienists, dermatologists, and dental surgeons, who can be called in for consultation in special cases.

But this is not all. The War Office has not only made provision for a specialist staff at each of its hospitals; it has also proceeded along the lines of the specialisation of institutions. This is a reform The Hospital has advocated in the past in connection with the Poor Law, and has urged the amalgamation of Poor-Law areas in order that it might be applied to existing institutions. The War Office has set aside hospitals or parts of hospitals for the treatment of neurological cases, orthopædic cases, cases of blindness, cases requiring artificial limbs, venereal diseases, dysentery, enteric-fever carriers, cerebro-spinal fever patients and carriers, cardiac cases, and cases of rheumatism. In addition, it has provided convalescent homes attached to groups of hospitals or to individual hospitals.

As regards the nursing staff an increase has also been made. Instead of a proportion varying from one nurse to seven patients, to one nurse to twelve patients, a uniform standard of about one nurse to five patients has been adopted.

Now comes the question, Are all these reforms to lapse at the end of the war? Are the infirmaries to be handed back to the Guardians and allowed to revert to their former state of comparative inefficiency due to the lack of a staff adequate to utilise them with the fullest efficiency? Are we to see expensive equipment lying idle for want of a staff trained to use it? Assuredly this will be the case unless radical changes in organisation and administration are made. The present Poor-Law system has had a long trial. It has failed in its hospital administration, in spite of repeated criticism, because it has not realised how complex medical work has become, and because the present Poor-Law areas are too small to provide economically the specialised staff and equipment necessary. The Poor-Law infirmary to-day is in the position of the voluntary hospital forty years ago, when the specialisation of medicine was still in its infancy. The reforms introduced by the War Office must not be lost. We must see that after the war the Poor-Law infirmaries are co-ordinated over large areas under authorities guided by experts in modern hospital administration.

